# Post-vasectomy Pain Syndrome: A Review of the Literature and Updated Treatment Algorithm

**DOI:** 10.7759/cureus.79592

**Published:** 2025-02-24

**Authors:** Michael Urbanski, Dyvon Walker, Jeffrey C Morrison, Majdee M Islam

**Affiliations:** 1 Urology, St. Louis University, St. Louis, USA; 2 Urology, University of Colorado-Anschutz, Aurora, USA; 3 Urology, Urology of St. Louis, St. Louis, USA

**Keywords:** chronic orchialgia, post vasectomy complications, post-vasectomy pain syndrome, scrotal pain management, spermatic cord block

## Abstract

Post-vasectomy pain syndrome (PVPS) affects a small but significant percentage of men following vasectomy. PVPS is characterized by persistent scrotal pain that disrupts daily activities and requires medical intervention. With hundreds of thousands of vasectomies performed annually in the US, and PVPS being a real and often devastating potential consequence, understanding its etiology and treatment options is crucial. Managing PVPS can be challenging, yet with thorough evaluation, it can be effectively addressed. It is imperative to undergo a comprehensive diagnostic process, including physical examination, urine studies, and imaging studies, to distinguish PVPS from other potential causes of scrotal pain.

Our objective was to review the literature on PVPS, highlight its etiology, and provide an updated evaluation and treatment algorithm. We conducted a comprehensive literature review using electronic databases via PubMed, which were searched from the start of publications until February 1, 2024. Studies including retrospective, prospective, observational, case-control, cohort, case series, and case reports were all eligible for review. Articles not published in English and conference abstracts were excluded from our review.

In our review, options for non-invasive treatments for PVPS include non-steroidal anti-inflammatory drugs (NSAIDs), tricyclic antidepressants (TCA), and anticonvulsants. Spermatic cord blocks are effective in diagnosing and managing chronic orchialgia, particularly when conservative treatments fail. Surgical interventions, including microsurgical denervation of the spermatic cord (MDSC), epididymectomy, vasovasostomy, and orchiectomy, are considered after exhausting non-invasive options. Various studies demonstrate the effectiveness of these surgical methods, highlighting their potential as treatment options depending on the individual case. An algorithmic evaluation method followed by a patient-specific treatment approach is key to managing PVPS, given its varied etiology and the differential effectiveness of treatment options. Understanding and addressing this complex condition is crucial to improving the quality of life for affected individuals.

In conclusion, an algorithmic evaluation method followed by a patient-specific treatment approach is key to managing PVPS, given its varied etiology and the differential effectiveness of treatment options. Understanding and addressing this complex condition is crucial to improving the quality of life for affected individuals.

## Introduction and background

In-office vasectomy is the most effective form of sterilization for men, during which, under local anesthesia, the vas deferens is disconnected to prevent sperm from entering the ejaculate. The vas deferens is skeletonized as fully as possible from the perivasal sheath, which is where much of the presumed nerve fibers are located, in order to decrease risk of any damage or inflammation to said nerves and other non-vas deferens structures. Various techniques are employed to avert recanalization, including the excision of a vas deferens segment, fulguration of the luminal ends, fascial interposition, and post-excisional suturing or clipping [1]. Post-vasectomy pain syndrome (PVPS) is a condition characterized by persistent scrotal pain not caused by testicular or epididymal infection, lasting over three months following a vasectomy, and is severe enough to disrupt daily activities and necessitates medical intervention [2,3]. Annually, over 500,000 vasectomies are performed in the US [4]. According to the 2012 American Urological Association (AUA) guidelines, about 1%-2% of men undergoing vasectomy will experience chronic scrotal pain affecting their quality of life, though a study by Auyeung et al. revealed that PVPS incidence post-vasectomy is approximately 5% for both scalpel and non-scalpel methods [5,6]. Persistent testicular pain is the most frequent vasectomy complication that can adversely interfere with quality of life for an extended period of time and requires varying degrees of medical treatment. Our goal, therefore, is to review the literature and provide an updated evaluation and treatment algorithm for the care of this difficult diagnosis.

## Review

Materials and methods

The literature was reviewed using electronic databases via PubMed (per institutional availability), which were searched from the start of publications until February 1, 2024. Studies including retrospective, prospective, observation, case control, cohort, case series, and case reports that discussed orchalgia, testicular pain, and post-vasectomy pain were all eligible for review as part of our inclusion criteria, and those most pertinent and significant were included. Articles not published in English and conference abstracts were not included in our review. As this is not a meta-analysis, all studies were not included in our discussion, and statistical analysis was not performed on the studies. 

Results

**Figure 1:**
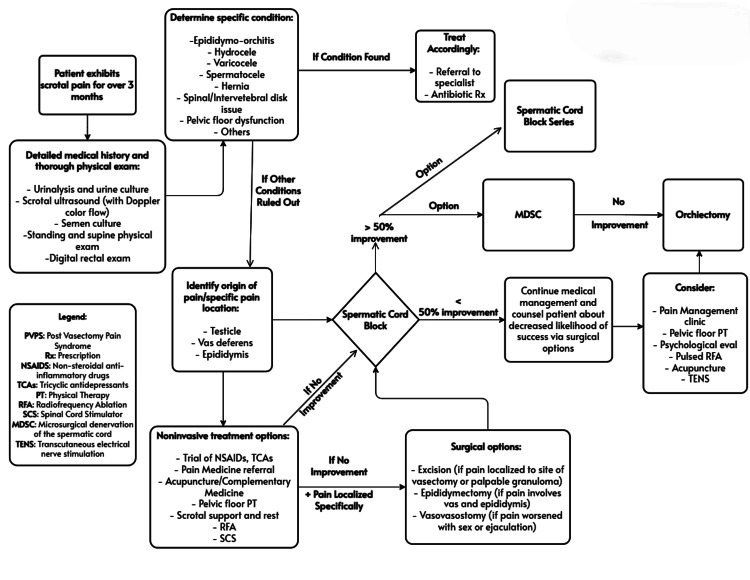
PVPS treatment algorithm PVPS: Post-vasectomy pain syndrome

Using our own experience, as well as our literature review, we developed an updated treatment algorithm for the evaluation, diagnosis, as well as the treatment of PVPS. We have included this below (Figure 1). Our results in more detail have been included in our discussion below. 

Discussion

The etiology of PVPS is unclear, with the condition often being considered to be multifactorial. Many etiologies have been described, including damage to spermatic cord structures, compression of nerves in the spermatic cord due to inflammation, back pressures caused by the blockage preventing sperm from advancing (epididymal congestion), an immune response to the vasectomy, perineural fibrosis, entrapment of nerves at the operative site, and painful sperm granulomas [7]. Antisperm antibodies that arise following the disruption of the blood-testis barrier may also trigger an organized immune response, as demonstrated by animal models [8]. These potential mechanisms, either individually or jointly, may ultimately lead to prolonged testicular or epididymal pain post vasectomy. The pain following a vasectomy typically lasts from several weeks to months and dissipates prior to a year [7].

Generally, it has been shown that the treatment approach for PVPS should implement all non-invasive/conservative options to relieve pain before proceeding to surgical alternatives if symptoms persist. It is also important to use non-surgical/conservative treatment options in order to evaluate for and rule out other potential causes of pain. The diagnosis of PVPS can only be considered after other etiologies for pain following vasectomy have been eliminated, which can include but aren't limited to an infection like epididymo-orchitis, hydrocele, varicocele, neuropathic pain, prostatitis, testicular neoplasm, hematoma, inguinal hernia, herniated disc or other back-related injury, intermittent testicular torsion, pelvic floor dysfunction, obstructing ureteral calculi, hip pathology, retroperitoneal tumors, and psychogenic causes [9-15]. Patients may present with unilateral or bilateral orchialgia, pain with ejaculation, intercourse, erection, physical activity, or worsened with pressure over the testis. When diagnosing PVPS, it’s crucial to ensure that the pain is not caused by other conditions that can otherwise individually be managed either conservative or surgically. This necessitates a comprehensive approach starting with a detailed history and physical examination at least three months post surgery. In medical history, it’s important to focus on duration of pain, type and location of pain (testicle, vas deferens, epididymis), and factors worsening pain such as ejaculation, intercourse, erection, physical activity, or pressure over testis. It’s also important to obtain information about any previous trauma or surgery in the pelvis, inguinal area or scrotum, current or previous genital infection, urinary complaints, back injury, analgesic use, and psychiatric disorders [9]. 

On physical examination of testis, epididymis, vas deferens, pelvis and abdomen, tender and/or full epididymis, tender proximal vas deferens, discomfort around the vessels of the cord, or palpable granuloma can be found [7]. Examination of scrotum should always begin on the unaffected side. During examination, testicular size and consistency, as well as presence of testicular mass, scars, hernia, epididymal abnormalities, and varicocele are evaluated. A scrotal ultrasound should be considered a gold standard and should be obtained to rule out other abnormalities. A digital rectal exam is prudent to rule out prostatic tenderness, hypertonicity and/or tenderness of pelvic floor musculature [11]. Since PVPS is a diagnosis of exclusion, the following tests should be performed before making the diagnosis: urinalysis, urine culture, semen culture, and expressed prostatic secretion culture [9]. Imaging studies such as CT abdomen and/or MRI of the spine are very useful when a patient reports backache or neurological symptoms [10,16]. This comprehensive evaluation ensures an accurate diagnosis, differentiating PVPS from other potential causes of chronic testicular pain.

Conservative (Non-procedural) Therapies for PVPS 

Non-invasive options involve various medications (pharmacotherapy) and other non-surgical modalities. Typically, patients approach the orchialgia first with non-steroidal anti-inflammatory drugs (NSAIDs), such as ibuprofen, meloxicam, and naproxen, for approximately two to four weeks. Other medications that may help alleviate the pain include tricyclic antidepressants (TCA) or anticonvulsants, such as gabapentin, pregabalin, amitriptyline, and clomipramine, that may be administered for four to six weeks. If neuropathic pain is suspected, these medications have been shown to improve symptoms of nerve pain or neuropathic pain [10,11]. Using narcotic medication over an extended period is not recommended for treating PVPS.

Other non-invasive options for PVPS are options like pelvic floor physical therapy versus acupuncture. Pelvic floor therapy may help patients with pain symptoms extended beyond typical PVPS symptoms that extend to other areas of their pelvic floor, which can include pain traveling up the spermatic cord into the groin area, or down towards the testicle. Although there is no literature noting acupuncture can alleviate pain from PVPS, it is a low-risk option that may help relieve some patients' pain symptoms [1].

It is practical, and should be common practice, for a spermatic cord block to be performed in order to confirm the spermatic cord nerve bundle as the primary pain source. Spermatic cord blocks can be pivotal in managing chronic orchialgia, especially when conservative treatments are ineffective and no clear reversible cause is evident. 

This procedure is usually performed using a 10-20 cc injection of a local anesthetic without epinephrine, plus or minus a steroid, targeting areas where Wallerian degeneration occurs in the spermatic cord, such as the cremaster muscle and the perivasal adventitious tissues [16]. If done with a steroid, often performed with 9 cc of local anesthetic and 1 cc of steroid mixed together. If, after the cord block, the patient has an improvement or there is resolution of their pain within a few hours to one or two days, then this increases the likelihood of the pain being from the nerves within the spermatic cord. 

However, these blocks not only aim to temporarily alleviate pain but can also relieve pain for a longer period of time and be diagnostic in nature. A spermatic cord block series, which typically uses bupivacaine and triamcinolone, aims to disrupt aberrant afferent peripheral pain signaling. The protocol involves administering a series of injections every two weeks, totaling four to five injections [12]. A retrospective study of 44 men who underwent this treatment showed that 70.5% experienced sustained relief, with 20.5% achieving complete pain resolution [13]. This procedure has shown promising results and presents itself as a potential treatment option for PVPS. It offers a minimally invasive and safe treatment option that can provide valuable diagnostic information and potential therapeutic benefit​, however, the long-term effect is still yet to be determined. 

From a diagnostic standpoint, when patients have a positive response from the block, this can indicate the spermatic cord/scrotum as the origin of the pain, and this response can be instrumental in determining the effectiveness of further interventions like microsurgical denervation of the spermatic cord (MDSC). As observed by Chaudhari et al., 62 out of 87 patients responded positively to a test dose, while 38 patients went on to have a MDSC, and 31 patients had complete resolution of their pain [9]. Therefore, if pain relief is achieved within a short period after the block, it strongly suggests that the pain originates from nerves within the spermatic cord, and denervation could be a viable option for long-term pain management.

Once all non-invasive pain relief options have been exhausted, surgical approaches to target the pain should be considered. The appropriate duration for non-invasive treatment before contemplating surgical intervention remains uncertain [14].

A variety of surgical alternatives exist, varying based on the presumed cause of the orchialgia after evaluation.

Surgical Interventions for PVPS

Excision of sperm granuloma: A sperm granuloma is a small nodule/mass that often forms at the site of the vasectomy due to sperm leaking out of the severed proximal end of the vas deferens and essentially solidifying/scarring. If the patient has a palpable sperm granuloma on a physical exam and the patient’s pain can be pinpointed to this, excision of the sperm granuloma should be considered [1]. A patient should be considered a potential candidate for this procedure if they are able to accurately identify their pain specifically to be specifically to the vasectomy site and to the site of the granuloma, and have this be reproduced on exam by the clinician [11]. The procedure typically is quite short, and the small mass is surgically removed under local anesthesia or over a short sedation course. Risks of this procedure, though rare, include persistent pain, bruising or swelling, and wound infection [11]. The advantage to this is if the patient’s symptoms are persistent after the procedure, one is still able to consider other treatment options. 

MSCD: If the patient reports at least 50% temporary pain relief following an appropriately applied spermatic cord block, denervation of the spermatic cord may be pursued. The pain relief indicates the origin of pain as the nerve bundle of the spermatic cord, and is considered an excellent predictor of success for treatment of symptoms with a MSCD [11]. The procedure involves microsurgically stripping the spermatic cord of all nerves and removal of all spermatic cord veins, with meticulous dissection and careful of preservation of the arterial vasculature and all lymphatic drainage, as well as consideration of ligation of the ilioinguinal nerve. It allows for identification of intral-scrotal pathology as the etiology behind the patient’s orchialgia. The procedure is performed most commonly in the operating room using a surgical microscope. Risks from the procedure can include persistent or worsening pain post procedure, formation of hydrocele, and potential injury to the arterial blood supply causing testicular damage. A notable disadvantage of the procedure is that it can potentially preclude a future vasovasostomy as the vasculature can be compromised to the vas deferens, though not common [11]. For patients that are good candidates for an MSCD, the success rates of MSCD can be quite high. There are multiple studies, including those by Chaudhari et al., Strom et al, Levine et al., and Ahmed et al., that confirm MSCD as an excellent option for targeting chronic post-vasectomy pain as the vast majority of patients report substantial pain relief, showing up to 77% success rates [9,17-19]. According to Tan et al., in a study of 27 patients with PVPS, no patient had a worsening numerical rating scale (NRS) score, which is used for documenting pain, after the MDSC [20]. However, in a research conducted by Kavoussi et al., it was found that men suffering from PVPS were more likely to experience unsuccessful outcomes with MSCD, especially when compared to men with idiopathic chronic orchialgia or chronic orchialgia following scrotal/inguinal surgery [21]. MDSC proves to be a fairly effective and beneficial method for treating PVPS, particularly when the pain affects various scrotal structures such as the testis, epididymis, and spermatic cord [22].

Epididymectomy: When the pain is localized to the posterior aspect of the testicle or the epididymis, performing an epididymectomy should be considered. This should only be considered if the discomfort can be specifically pinpointed on exam to the epididymis itself and reproduced on exam during palpation as well, without pain in other areas of the testicle or the spermatic cord. The epididymectomy can be a straightforward procedure, but surgeons must ensure no damage is performed to the rest of the vasculature of the spermatic cord due to the proximity in anatomy. Other risks to this procedure include persistent or worsening pain, hydrocele formation, and damage to the testicle. Again, a disadvantage of this procedure is that if the patient's pain does not resolve following this procedure, a vasovasostomy is not possible as vas deferens is transected along with the epididymis. Interestingly, a study by Chung et al. involved patients with PVPS undergoing an epididymectomy with a simultaneous hyaluronic acid and carboxymethyl acid injection to prevent fibrosis, which resulted in even better pain relief results [23]. Additionally, Lee et al. studied 50 patients with PVPS who underwent an epididymectomy or vasectomy reversal and observed no significant difference between these two procedures regarding reduction in pain or the satisfaction with surgical outcome [24]. That is why specific patient characteristics may impact the choice of procedure. A study by Hori et al. evaluating 53 patients between 1994 and 2007 (mean follow up of 7.4 years) who had undergone epididymectomy mostly (85%) for post-vasectomy pain reasons exhibited significant improvements in pain scores, with 93.3% reporting less or no postoperative pain [25].

Vasovasostomy: If the patient has no concern for regaining potential fertility in the future, a vasectomy reversal, or vasovasostomy, can be an effective treatment to PVPS. Candidates for this treatment option may feel pressure and congestion in their scrotum unilaterally or bilaterally, especially with ejaculation. The goal of the procedure is to decrease the congestion and increased theoretic pressure due to the obstruction at the vas deferens’s proximal stump. Success rates can be quite high with good patient selection, with Horovitz et al. reporting in their single-center study upwards of 93% of patients with pain relief from the procedure [26]. Also in this study, 79% of patients had a durable positive response after the procedure and 93% said they would undergo the same operation again. Potential risks to this option include a persistent or worsening pain, and possible return of initially undesired fertility requiring usage of anticontraceptives [1]. However, patients who have a vasovasostomy may still undergo other surgical treatment options if their pain persists. An important factor to consider with this option is the lack of insurance coverage for a vasovasostomy done for fertility reasons or for PVPS, which can prove to be cost-prohibitive for some patients. A vasectomy reversal can be considered an appropriate surgical therapy after non-surgical treatments have failed in patients that have more congestive type pain symptoms such as bilateral testicular pain that worsens after ejaculation. For these patient groups, the success rate of achieving a substantial decrease in pain ranges from 69% to 100%. Conversely, MDSC may be a viable option for patients with PVPS who experience more persistent, one-sided, or nerve-related pain [27,28].

Orchiectomy: Removal of the testicle and spermatic cord is a drastic measure and should be considered a last resort once all surgical and medical options have been exhausted for patients with persisting chronic orchialgia. A radical inguinal orchiectomy can be considered over a simple scrotal orchiectomy due to some data reporting higher potential for complete pain relief of postoperative pain [28]. Success rates for substantial reduction in pain after this surgery was reported between 20%-75% [27]. The risks to this procedure include a theoretical potential decrease of testosterone, and rare persistent pain. Additionally, some patients may still have pain once the testicle is removed, similar to phantom-limb pain following amputation. The mechanism of this pain is unclear, and the pain can be located in the contralateral testicle [29]. This invasive approach requires proper patient counseling because of the possible complications such as hypogonadism, sexual implications, and psychological alterations [30]. 

## Conclusions

Dealing with PVPS can be a challenging experience for both the clinician and the patient. Therefore, thorough evaluation of the causes, diagnosis, and various treatment possibilities of PVPS is essential in enhancing the patients' overall quality of life. In case of failure of pharmacological and non-surgical treatment, many surgical options are available to patients with PVPS. In regards to surgical options, if the patient has a palpable painful granuloma this can be excised; otherwise, a spermatic cord block should be considered because it is a low risk option that can direct the treatment plan toward a microdenervation of the spermatic cord if successful and also allows the patient to consider other surgical options if unsuccessful. Epididymectomy is a good treatment option for a cyst, granuloma, or mass on the epididymis. If the patient is concerned about sterility, it is best to avoid a vasovasostomy. For patients exhibiting symptoms more indicative of congestion, vasectomy reversal can be considered as a treatment approach. An orchiectomy should always be the very last option taken due to the potential hormonal effects as well as the severity of the procedure.

While it can be appropriately prudent to defer a surgical solution to post-vasectomy pain, it is evident that effective solutions to resolve chronic orchialgia following a vasectomy refractory to non-invasive therapy often are surgical ones. It is important to remember that each person experiencing PVPS has a unique anatomy and thus a unique etiology, so the optimal treatment options and their effectiveness is variable for every patient.
